# A CHECKLIST-BASED INTERVENTION TO SUPPORT FAMILY CAREGIVERS IN PRIMARY CARE

**DOI:** 10.1093/geroni/igad104.1881

**Published:** 2023-12-21

**Authors:** Catherine Riffin

**Affiliations:** Weill Cornell Medicine, New York City, New York, United States

## Abstract

Family caregivers commonly accompany older adults to their routine medical visits, but caregivers’ own needs and concerns are not systematically identified or addressed in clinical practice. Collaborative Healthcare Encounters with Caregivers (CHEC) is a checklist-based intervention that uses a prioritization approach to help caregivers efficiently identify and communicate their most salient concerns to primary care providers. This presentation will report findings from a pilot randomized controlled trial of N=37 patient-caregiver dyads who were randomized to CHEC (N=21) or usual care (n=16). The goals of the pilot trial were to evaluate CHEC’s impact on medical visit duration and communication (using time-stamped audio-recordings and a structured coding system) and caregivers’ perceptions of CHEC’s utility (using questionnaires and interviews). Total visit duration was comparable across treatment groups (CHEC: 29.3 minutes vs. UC: 32.8 minutes; p=0.55), but a greater proportion of the visit time was spent addressing caregivers’ concerns among CHEC group participants, as compared to usual care participants (25% vs. 6%; p<.001). In post-visit interviews, caregivers commented that CHEC was useful in helping them to prepare for the visit by thinking about their questions in advance and prompting providers to acknowledge their concerns. Findings from this study explicitly address the RAISE Act’s recommendation for integrating evidence-based caregiver assessment instruments into clinical care delivery (Outcome 2.2) and indicate CHEC’s potential as an efficient, clinically feasible intervention that can support greater inclusion of caregivers in primary care discussions.

